# A Tutorial on Hunting Statistical Significance by Chasing *N*

**DOI:** 10.3389/fpsyg.2016.01444

**Published:** 2016-09-22

**Authors:** Denes Szucs

**Affiliations:** Department of Psychology, University of CambridgeCambridge, UK

**Keywords:** replication crisis, Type I error, false positive error, null hypothesis significance testing (NHST), bias and data dredging, p-hacking, N-hacking

## Abstract

There is increasing concern about the replicability of studies in psychology and cognitive neuroscience. Hidden data dredging (also called p-hacking) is a major contributor to this crisis because it substantially increases Type I error resulting in a much larger proportion of false positive findings than the usually expected 5%. In order to build better intuition to avoid, detect and criticize some typical problems, here I systematically illustrate the large impact of some easy to implement and so, perhaps frequent data dredging techniques on boosting false positive findings. I illustrate several forms of two special cases of data dredging. First, researchers may violate the data collection stopping rules of null hypothesis significance testing by repeatedly checking for statistical significance with various numbers of participants. Second, researchers may group participants *post hoc* along potential but unplanned independent grouping variables. The first approach ‘hacks’ the number of participants in studies, the second approach ‘hacks’ the number of variables in the analysis. I demonstrate the high amount of false positive findings generated by these techniques with data from true null distributions. I also illustrate that it is extremely easy to introduce strong bias into data by very mild selection and re-testing. Similar, usually undocumented data dredging steps can easily lead to having 20–50%, or more false positives.

## Introduction

It is increasingly acknowledged that psychology, cognitive neuroscience and biomedical research is in a crisis of producing too many false positive findings which cannot be replicated (Ioannidis, 2005; Ioannidis et al., 2014; Open Science Collaboration, 2015). The crisis wastes research funding, erodes credibility and slows down scientific progress. Here I systematically review two potential major sources of false positive production: the neglect of hidden multiple testing in studies both in terms of case (participant) and variable numbers. First, researchers may test a sample for statistical significance and then re-run significance tests after adjusting case (participant) numbers (Barnett and Lewis, 1994; Wilcox, 1998; Wagenmakers, 2007; Simmons et al., 2011; Bakker et al., 2012; Bakker and Wicherts, 2014; Simonsohn et al., 2014). Second, researchers may run significance tests for multiple, *ad hoc* selected independent grouping variables (Meehl, 1967; Simmons et al., 2011; Francis, 2013; Silberzahn and Uhlmann, 2015). Both of these phenomena can vastly inflate false positive Type I error. While many researchers may be in principle conscious of the dangers of manipulating case and variable numbers in analyses, they may not appreciate just how easily such practices lead to generating a large number of false positive results. In order to provide a better intuition for such Type I error inflation and to provide a reference point to avoid, recognize and criticize these mistakes, here I illustrate the impact of data dredging steps through a number of simulations which can be understood easily visually.

Data dredging techniques aim to achieve statistically significant *p*-value levels and hence, they are also called ‘p-hacking’ (e.g., Bruns and Ioannidis, 2016). A lot suggests that unintentional and intentional data dredging is a very likely contributing factor to the overwhelmingly positive results published in many sciences (Ioannidis and Trikalinos, 2007). Psychology especially seems to be affected by this as positive results are about five times more likely in this discipline than in some physical science areas (Fanelli, 2010). In general, social sciences are 2.4 times are more likely to generate positive reports than physical sciences (Fanelli, 2010). In addition, the prevalence of positive results seems steadily increasing during the past decades, especially in social and some biomedical sciences which suggests that the prevalence of data dredging is increasing (Fanelli, 2012).

In this paper I deal with special cases of what Simmons et al. (2011) called ‘researcher degrees of freedom,’ or put otherwise, special factors behind what Ioannidis (2008) termed ‘the vibration ratio.’ ‘Researcher degrees of freedom’ refers to undisclosed flexibility in data analysis, i.e., to the fact that researchers have many potential analysis solutions to choose from and many of their choices (and their rationale) are undocumented. ‘Vibration ratio’ refers to widely varying effect sizes on the same association in response to different analytical choices. In general, the more analytical choices researchers try latently, the higher is the chance of hitting some spurious statistically significant findings because of the multiple testing problem (see below). My intention here is to systematically illustrate some easy to implement and therefore perhaps typical data dredging techniques which can highly inflate Type I error and thus, of course, can result in false positive publications with statistically significant findings. In response, these techniques will hopefully become easier to recognize, prevent and criticize.

Sequential re-analysis of study data is a well-known potential contributor to an excess of statistically significant findings (Demets and Gordon-Lan, 1994; Goodman, 1999). Similarly, cherry-picking variables with statistically significant relationships is another well-known potential contributor to spurious findings (Bakan, 1966; Meehl, 1967; Waller, 2004; Bruns and Ioannidis, 2016). In this sense, here I illustrate these two special cases of p-hacking in detail. These special cases could be termed ‘N-hacking’ because they latently manipulate case and variable numbers with the intention of p-hacking. I use visualization of simulation data because this approach has been thought to be helpful for better understanding of statistical phenomena (see Sellke et al., 2001) and visualization is thought to increase the understanding of mathematical functional data in general (Gleason and Hughes Hallett, 1992). However, note that claims about the efficacy of visualization require further empirical validation and as such are worthy goals for future study on their own.

### The Multiple Testing Problem

During Null Hypothesis Significance Testing (NHST) researchers aim to reject a null hypothesis (the null; often signified as *H*_0_) which assumes that there is no difference between experimental conditions and/or groups on some measure. Researchers compute a test statistic from their data and examine the associated *p*-value (p). The *p*-value is the probability of having a test statistic as extreme or more extreme than the one computed from the data given that the null is true. NHST controls the long run probability of false positive (Type I) errors through setting α, a pre-determined critical threshold parameter, the long run probability of finding a statistically significant test outcome when the null hypothesis is in fact true. The NHST framework assumes that the null is too unlikely to be true if *p* ≤ α (note that this is a false assumption from the Bayesian point of view but discussing this is out of the scope of this paper; see e.g., Pollard and Richardson, 1987; Ioannidis, 2005). In such a case the null is rejected and the alternative hypothesis (often signified as *H*_1_) of having a non-null effect is accepted. NHST can also control the long run probability of discovering true effects provided that their effect size is known and the sample size can be adjusted. This probability is called ‘power’ and it is the complement of the long-run probability of not discovering true effects if they exist (Type II error), called β. Hence, Power = 1-β (Neyman and Pearson, 1933).

**FIGURE 1:**
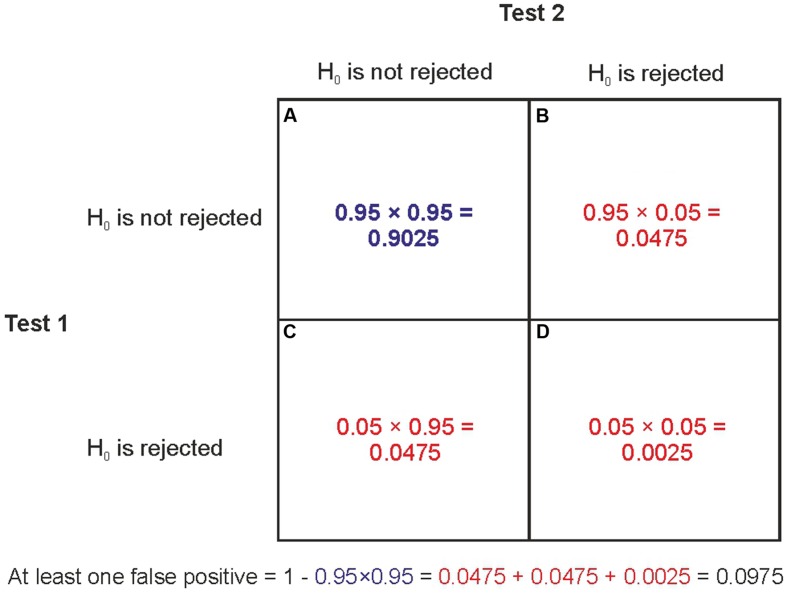
**The computation of family-wise error rate.** Let’s assume that the null hypothesis is true and we run two independent significance tests with α = 0.05. There are four possible outcomes: **(A)** The probability that none of the tests can reject the null is 0.95 × 0.95. **(B)** The probability that the first test does not reject the null but the second does reject it is 0.95 × 0.05. **(C)** The probability that the first test rejects the null but the second test does not reject it is 0.05 × 0.95. **(D)** The probability that both tests reject the null is 0.05 × 0.05. The family-wise Type I error rate is the probability that at least one of the tests rejects the null. This is the probability of the complement of **(A)**. That is, the summed probability of all other possible outcomes besides **(A)**. Put more technically, the complement of **(A)** is the probability of the union of **(B–D)**: 0.0475 + 0.0475 + 0.0025 = 0.0975. Because **(A–D)** represent all possible outcomes their probabilities sum to 1. Hence, the complement of **(A)** can also be computed as 1-0.95^2^ = 0.0975.

In the overwhelming majority of studies α is set to 0.05 which means that researchers expect that only 5% of studies with true null effects would turn up statistically significant findings. The core problem in all the data dredging problems illustrated here is the well-known multiple comparison problem of NHST: if we repeatedly test for statistical significance in multiple tests with a certain α level than the Type I error rate becomes inflated. If the repeated tests concern independent data sets where the null is true than the probability of having at least one Type I error in k independent tests, each with significance level α, is α_TOTAL_ = 1-(1-α)^k^. For example if *k* = 1, 2, 3, 4, 5, and 10 than α_TOTAL_ is 5, 9.75, 14.26, 18.55, 22.62, and 40.13%, respectively (e.g., Curran-Everett, 2000). A group of statistical tests which are somehow related to each other can be defined as a ‘family of comparisons’ and the probability that this family of comparisons contains at least one false positive error is called the family-wise error rate (FWER), defined as α_TOTAL_ above. **Figure 1** illustrates the logic behind computing the family-wise Type I error rate.

## Hacking the Number of Cases/Participants in A Sample

While many researchers may be conscious of the dangers of multiple testing it is often less appreciated that not adhering to the data collection stopping rules of NHST also inflates Type I error rate due to the multiple comparison problem. Data collection stopping rules are violated when, after initial significance testing researchers add new participants to the sample, drop some participants and/or swap some participants for new participants either randomly or with some (perhaps unconscious) selection bias and then re-run tests to repeatedly check for statistical significance. Such techniques can be used during sequential data collection and/or when dealing with supposedly outlier cases (Barnett and Lewis, 1994; Wilcox, 1998; Wagenmakers, 2007; Bakker et al., 2012; Francis, 2013; Bakker and Wicherts, 2014; Simonsohn et al., 2014).

For example, if we collect 40 data sets about a true null phenomenon with 16 participants/cases in each and run tests with α = 0.05 then about two tests will turn up statistically significant results by chance alone. Now, after this first run of tests we may not be fully satisfied with the results and may think that we did not have enough power in our previous series of tests. So, we may decide to add another participant to all the samples and re-run the tests. If we use α = 0.05 then we may again find two statistically significant results in this new series of tests, that is, the Type I error rate is 5% as before. However, by adding new participants to the samples and retesting for statistical significance we also exposed ourselves to the multiple comparison problem.

**FIGURE 2:**
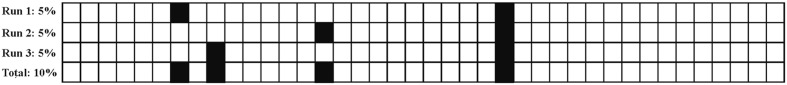
**Illustrating how repeated testing of non-independent data sets can lead to the accumulation of false positive Type I errors.** The boxes stand for statistical tests run on true null data samples. The empty boxes denote tests with non-significant results. The filled boxes denote tests with statistically significant false positive results. First, we run 40 tests with α = 0.05 and 5% of them (two tests) will come up statistically significant (Run 1). Second, we slightly change the data sets and re-run the tests (Run 2). While again 5% of tests will come up statistically significant, these will not necessarily be the same two data sets as before. A similar phenomenon happens if we slightly change the data again and re-run the tests (Run 3). In the example, the consequence of repeated testing of altered data is that the total Type I error rate in terms of the 40 data sets will be 10% rather than 5%.

An important point is that if we add new participants to our original sample, the repeated tests will not be run on independent samples because most of the participants are the same in all samples. Nevertheless, it is not guaranteed that the same data sets will provide statistically significant findings during both the first and the second series of tests because adding additional participants changes some parameters of the data sets. So, data sets with previously statistically significant findings may now provide non-significant findings and vice-versa. That is, while the Type I error rate is 5% in both series of tests, different data sets may turn out to be false positives in both cases. This means that at the end of the second test series more than 5% of the 40 data sets may have provided statistically significant findings if we consider both 2 × 40 test runs (see **Figure 2**).

The violation of stopping rules may happen frequently in real research for various reasons. Below I demonstrate the extreme impact of some of these techniques on generating false positive results on simulated data even when all data is coming from a null distribution. (All simulations were run in Matlab 2015b^1^).

### Adding Participants to Samples and Re-Testing

In real world experimentation researchers may initially collect (pilot) data from a relatively small number of participants and test results for statistical significance. If the results are not statistically significant but they are fond of the experimental idea researchers may decide to add some more participants to the sample and re-test for statistical significance. Tests for statistical significance may be repeated numerous times after adding more and more participants to the sample. Researchers often perceive this procedure as legitimate means to increase power through increasing the sample size and may not be conscious of the fact that they are violating the basic sampling rules of NHST: If they repeatedly check for statistical significance after adding each individual participant to the sample they quickly accumulate Type I error. Hence, the ‘cumulative’ Type I error across all the statistical tests done will be much higher than 5%.

**FIGURE 3:**
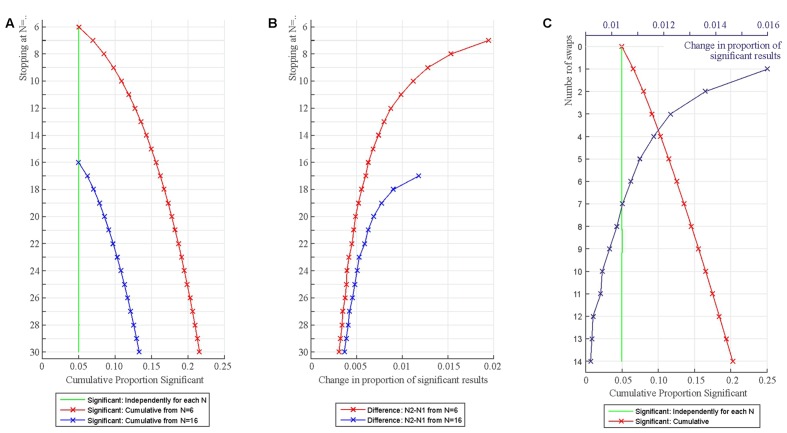
**Increase in statistically significant results when adding additional participants to samples **(A,B)** and when randomly swapping participants for new ones **(C)**.** **(A)** The figure shows the proportion of false positive significant results independently for each *N* (green line) and when considered cumulatively up to a particular *N* (red and blue lines). **(B)** The rate of increase in statistically significant test outcomes represented in Panel A from a particular N to N+1. **(C)** Increase in statistically significant results when swapping one randomly chosen participant in the sample for another one. The number of swaps is represented on the *Y*-axis (0–14 swaps). The green line shows the proportion of statistically significant results independently for each test run. The red line shows the proportion of statistically significant results cumulatively for each test run. The blue line shows the rate of increase in statistically significant results from one swap to the next.

**Figure 3A** demonstrates the accumulation of Type I error with repeated testing. First, one million data sets were simulated with *N* = 6 from a standard normal distribution (*M* = 0; *SD* = 1). One-sample *t*-tests with α = 0.05 determined whether the sample mean was zero. After this, another participant was added to each sample (*N* = 7) and the tests were re-run. This process was repeated until *N* reached 30. As shown, we have 5% false positives as expected when samples with each *N* are tested independently. However, the cumulative number of false positives is increasing rapidly and exceeds 10% just after adding four more participants (*N* = 10) and 15% after reaching *N* = 16. In practice, if researchers carry out the above procedure and they detect a statistically significant result they may decide to stop and publish that result. As the simulations demonstrate researchers actually have a fairly good chance of detecting a statistically significant finding even when the null is true.

It is worth noting that the increase in the proportion of statistically significant findings is the fastest at smaller Ns because with smaller samples adding one additional data point can exert a relatively large effect on the overall parameters of a data set (**Figure 3B**). This can be appreciated if we observe what happens when we start testing with a larger number of participants (from *N* = 16; blue line in **Figure 3A**). In this case Type I errors accumulate just as well as when starting at *N* = 6 but the rate of increase of Type I errors is smaller (**Figure 3B**). This is because adding one additional data point exerts a smaller relative effect on the overall parameters of a larger than a smaller data set. Hence, there is less variability in which data sets show statistically significant findings after repeated testing. This also means that larger Ns are more resistant to false positive generation (but do not protect against it), so running larger studies can be recommended from this point of view. For this reason, Simmons et al. (2011) recommended that studies should have at least 20 cases per relevant statistical cell. Also note that larger Ns also boost power which is often very low in psychological and neuroscience research (Sedlmeier and Gigerenzer, 1989; Rossi, 1990; Button et al., 2013).

### Swapping Participants for New Participants

Another way of violating stopping rules is removing some participants deemed to be too noisy, to be ‘outliers,’ or ‘failed experiments’ and replace them with randomly selected new ones (without increasing the overall participant numbers as above). Of course, sometimes such participant replacement is inevitable (in genuinely failed experiments). However, each new replacement provides another chance for Type I error even if there is absolutely no bias in removing and swapping participants.

**Figure 3C** demonstrates the accumulation of Type I error when swapping a randomly selected participant from the original sample for a new participant and retesting. First, one million data sets were simulated with *N* = 16 from a standard normal distribution (*M* = 0; *SD* = 1). One-sample *t*-tests with α = 0.05 determined whether the sample mean was zero. After this, a *randomly* selected participant was deleted from the sample and replaced with another participant also generated from the standard normal distribution keeping *N* = 16 and the tests were re-run (*Y* = 1 in **Figure 3C**). Fourteen swaps were generated (*Y* = 1 to 14 in **Figure 3C**).

As expected, we have 5% false positives when samples with each *N* are tested independently. However, the cumulative number of false positives is increasing rapidly and exceeds 10% just after four swaps and 15% after nine swaps (*Y* = 4 and *Y* = 9 in **Figure 3C**). The rate of increase in the proportion of statistically significant findings is the fastest for the initial 1–5 swaps because some of the randomly created data sets are easier to move into the ‘statistically significant direction’ than others.

Researchers should be conscious of this implication and should not liberally remove ‘suspected’ outliers when such removal cannot be justified clearly. Removal and swapping of participants is a particular concern in neuroscience experiments where it may be easy to refer to physiological noise as a justification for the removal of participants. As a minimum, all removals and swaps should be documented. When removal cannot be justified clearly confidence intervals and effect sizes should be presented with and without the removed participants added to the sample.

### Culling ‘Outliers’ without Replacement

In section “Swapping Participants for New Participants” (**Figure 3C**) supposed ‘outliers’ to be removed from the sample were selected completely at random without any bias. However, researchers can easily have some slight unconscious or conscious biases which can infiltrate the data and these can also lead to substantial increase in Type I errors. For example, it may happen that participants are judged ‘outliers’ if they are dissimilar to expectations and such outliers are then replaced with new data values. Such practices may still be the consequence of unconscious bias but they may also constitute outright fraud if they are done systematically through many studies. As illustrated below, such bias can be introduced into the data in very delicate ways and still have a major impact on the number of false positive findings.

**FIGURE 4:**
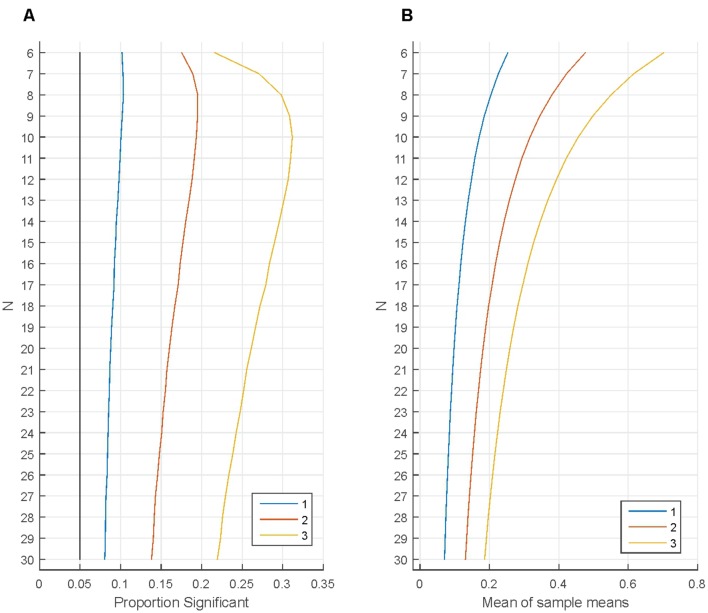
**Removing the least fitting participants from the sample without replacing them.** **(A)** The proportion of statistically significant findings independently for various numbers of participants (‘*N*’ on the vertical axis). The black line indicates the proportion of statistically significant findings when testing the original *N* number of participants. The other lines indicate the proportion of statistically significant findings when removing 1, 2, or 3 participants with the most negative data points from the sample. **(B)** Illustrates how the mean of sample means changes when removing 1, 2, or 3 participants with the most negative data points from the samples.

**Figures 4A,B** demonstrate the effect of removing the least fitting participants from the sample without replacing them. First, 1 million samples were generated from a normal distribution for each *N* (*M* = 0; *SD* = 1). One sample t-tests tested whether the sample means were zero (α = 0.05). As the black line indicates, the proportion of Type I errors was 5%. After the initial tests the participant with the most negative value was removed from the sample and the tests were re-run. As shown, the proportion of Type I errors increased noticeably, for small Ns (*N* = 6–12) the proportion of Type I error doubled. When one additional participant (with the remaining most negative value) was removed from each sample the proportion of Type I errors increased dramatically, becoming larger than 13% for all Ns studied and when a third participant was removed from each sample the proportion of Type I errors exceeded 25% for Ns between 7 and 23. This range of participants is extremely typical in psychological research.

### Culling Outliers with Replacement

**FIGURE 5:**
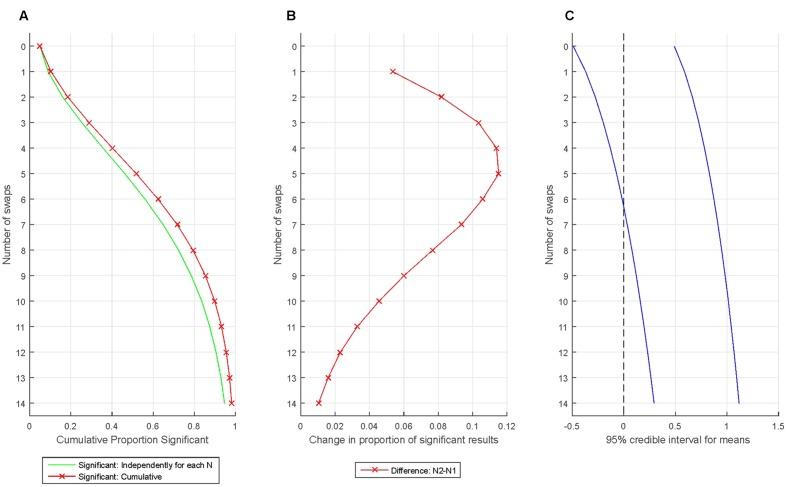
**Increase in statistically significant results when introducing very mild bias and re-testing.** **(A)** The proportion of statistically significant findings independently (green line) and cumulatively (red line) for various swaps. **(B)** The rate of increase in the proportion of statistically significant findings from one swap to the next. **(C)** The change introduced into the sample mean by the biasing process is illustrated by plotting the 95% credible interval for the sample means (assessed from the simulation).

**Figures 5A–C** demonstrate the extreme fast accumulation of Type I error when we bias results by consecutively removing the participants least fitting our expectations and replacing them with randomly selected new participants. First, one million data sets were simulated with *N* = 16 from a standard normal distribution (*M* = 0; *SD* = 1). One-sample *t*-tests with α = 0.05 determined whether the sample mean was zero. After this, the participant with the most negative data point was deleted from the sample and replaced with another participant’s data generated randomly from the standard normal distribution. Fourteen swaps were generated. Note that the above procedure replaces the old data with in principle unbiased values generated completely at random from the standard normal distribution. However, the continuous ‘culling’ of ‘outliers’ still makes a massive impact on the sample mean: Just after swapping two participants the proportion of false positives is nearly 20% even when considering tests independently for that particular test run! Then, after swapping just 4 out of 16 participants the proportion of false positives reaches 40% and after swapping five participants (less than 1/3 of the sample), the cumulative false positive rate passes 50%!

## Hacking the Number of Independent (Grouping) Variables

### Subgroup Testing Using Weakly Correlated Grouping Variable

It may happen that researchers’ main hypothesis does not work out, or that study objectives were only very fuzzily defined to start with. However, to the rescue, researchers may have several potential grouping variables for their data. Groups can then be formed after the study was run and groups can be compared to see whether there are statistically significant differences between groups along the *ad hoc* defined grouping variables. This process is a fairly refined form of data dredging (splitting *N* into two; e.g., schizophrenic and non-schizophrenic) and can easily be camouflaged as testing an *a priori* hypothesis if group membership can be justified with *post hoc* arguments. In fact, it is easy for researchers to rationalize *post hoc* that the study *could have been* planned in the way as it was ultimately written up for publication.

**FIGURE 6:**
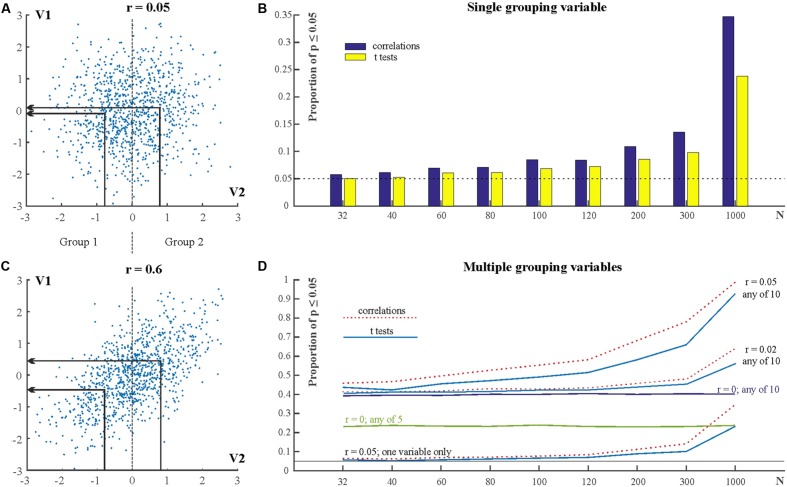
**Using *ad hoc* grouping variables along weakly or non-correlated variables.** **(A)** Scatterplot for two variables (V1 and V2) with *r* = 0.05. Groups are defined by V2. The group means on V2 and V1 are indicated by the arrows. **(B)** Scatterplot for two variables with *r* = 0.05. **(C)** The proportion of statistically significant results in correlations and *t*-tests for various sample sizes. **(D)** The proportion of statistically significant results when using multiple independent (grouping) variables. See explanation in text.

In the above situation many more than 5% of tests can be expected to reach statistically significant levels even when the grouping variable is only very weakly correlated with the dependent variable. **Figure 6A** shows two very weakly correlated variables (V1 and V2) with a correlation coefficient of *r* = 0.05 (*N* = 1000). For comparison, **Figure 6B** shows two strongly correlated variables with *r* = 0.6. We can consider V2 as grouping variable and form two groups. Group 1 is defined by V2 ≤ 0 and Group 2 is defined by V2 > 0 (For example, Group 1 may have low IQ scores and Group 2 may have high IQ scores). That is, the sample size in the two groups is N/2. We can then run independent sample *t*-tests to compare the means of V1 in Groups 1 and 2. The above procedure was simulated by generating 10,000 samples for various Ns. **Figure 6C** shows the proportion of statistically significant results for different values of *N* (e.g., if *N* = 32 then the sample size in both groups is 16). It can be seen that as *N* is increasing the proportion of statistically significant results is slightly increasing above the expected 5% level. However, when *N* is large (*N* = 1000), the proportion of statistically significant results increases dramatically and exceeds 20%.

The explanation for the very high proportion of significant findings when *N* is high has been known since a long while (Meehl, 1967): NHST test statistics are typically computed as the ratio of the relevant between condition differences and associated variability of the data weighted by some function of the sample size [difference/variability × f(sample size)]. The *p*-value is smaller if the test statistic is larger. That is, the larger the difference between conditions and/or the smaller is variability and/or the larger is the sample size the larger is the test statistic and the smaller is the *p*-value. Consequently, by increasing sample size enough it is guaranteed that the null can be rejected even when effect sizes are very small. The above simulation exemplifies exactly this situation: forming subgroups along the weakly correlated *ad hoc* grouping variables leads to small group differences in the dependent variables. These small group differences will then inevitably reach significant levels once *N* is high enough.

Importantly, the simulation is also ecologically valid in that it is practically inevitable to have at least some weak correlation between any psychological variables. This is because psychological phenomena are very complex reflecting the contribution of several interacting (latent) factors (Meehl, 1967; Lykken, 1968). Hence, if we select any two variables related to these complex networks most probably there will be some kind of at least remote connection between them. Second, unlike in physical sciences it is near impossible to control for the relationship of all irrelevant variables which are correlated with the variable(s) of interest (Rozeboom, 1960; Lykken, 1968). Hence, if we select any two variables at random it is likely that their correlation will be different from zero. Now, if we also have a large sample size this state of affairs will inevitably lead to the situation exemplified in the simulation!

### Subgroup Testing Using Multiple Potential Weakly Correlated Grouping Variables

The above section exemplified a situation where there was only one potential grouping variable. However, researchers may have more than one potential grouping variable, especially nowadays when larger and larger databases are available as we are entering the era of big data (Khoury and Ioannidis, 2014). Increasing both the number of potential grouping variables and the number of participants have tremendous potential for inflating Type I error. **Figure 6D** shows the outcomes of simulations similar to the one in section “Subgroup Testing Using Weakly Correlated Grouping Variable” but which included 5 or 10, rather than only one potential grouping variable. 10,000 samples were simulated for each *N*. Each simulation generated a dependent variable (V1) and 10 potential grouping variables (V2–V11). In the first series of simulations V1 had correlation *r* = 0.05 with each of the other individual variables (V2–V11). Groups were defined according to each of V2–V11. That is, first Group 1 was defined by V2 ≤ 0 and Group 2 was defined by V2 > 0. Independent sample *t*-tests compared the means of V1 in Groups 1 and 2. After this V3 served as grouping variable, then V4 served as grouping variable, and so on. The bottom lines (labeled ‘*r* = 0.05; one variable only’) in **Figure 6D** show the proportion of statistically significant test outcomes for each *N* for an individual grouping variable. These proportions are the same as in **Figure 6C.** In addition, **Figure 6D** also shows the cumulative proportion of statistically significant results for *all* grouping variables for various situations (*r* = 0; *r* = 0.02; *r* = 0.05; cumulative proportions for 5 and for 10 variables). This cumulative proportion is the family-wise Type I error rate, the probability of getting at least one statistically significant result when we test the samples on *any* of the grouping variables. It can be seen that just with *N* = 32 this probability is already larger than 0.4 for *t*-tests and if *N* = 1000 then it is practically guaranteed that we can detect some significant group differences along a potential grouping variable. In fact, even when the grouping variable and the dependent variable are completely uncorrelated (*r* = 0) the false positive Type I error rate is 22% with five potential grouping variables (see line marked: ‘*r* = 0; any of 5’ in **Figure 6D**) and 40% with 10 potential grouping variables see line marked: ‘*r* = 0; any of 10’ in **Figure 6D**).

It is worth pointing out that knowing about a correlation of *r* = 0.05 is typically irrelevant in practical terms. This is because if we look at **Figure 6A** it is obvious that such a correlation means that we cannot really predict anything about a variable (e.g., V1) if we know the other, correlated one (V2).

While the above issues are known since at least the 1960s (Rozeboom, 1960; Meehl, 1967; Lykken, 1968) they are very often neglected perhaps because it is different to know about these problems *in principle* and in practical terms.

## Discussion

Here, I have illustrated in detail two particular forms of data dredging: the hidden manipulation of the number of cases (participants) tested and the number of grouping variables exploited in studies. If researchers judge sequential testing necessary then in principle FWER multiple testing correction methods (Shaffer, 1995 and Nichols and Hayasaka, 2003 for review) and False Discovery Rate (FDR) control could be used to deal with these problems and excellent reviews are available on these (see Benjamini and Hochberg, 1995; Curran-Everett, 2000; Benjamini and Yekutieli, 2001; Nichols and Hayasaka, 2003; Bennett et al., 2009; Benjamini, 2010; Goeman and Solari, 2014). There is also a large directly relevant literature of controlling Type I error rates in repeated interim analyses in sequential clinical trials through the study of ‘alpha spending functions’ (Anscombe, 1963; Demets and Gordon-Lan, 1994; Whitehead, 1999; for review see Whitehead, 1997; Shih and Aisner, 2016). A more radical solution is to abandon the NHST paradigm and use Bayesian models if possible which can update the posterior values for model parameters after new (sequential) data comes in without encountering the multiple testing problem (MacKay, 2003; Sivia and Skilling, 2006; Gelman et al., 2014). Similarly, Bayesian hierarchical models may also be able to cope with situations where several multiple tests are inevitable (Gelman et al., 2012).

However, most importantly, because of the hidden (unpublished) nature of data dredging manipulations such manipulations primarily need a ‘cultural solution’: researchers have to take the problems reviewed here seriously, document repeated testing steps whenever they may happen and treat them somehow. Currently many researches may not even be conscious of the very serious Type I error inflation caused by repeated testing and regrouping variables and may consider these processes as legitimate ways to ‘test whether power is adequate to publish the results’ so that testing can be stopped as soon as possible and research funding can be used for other experiments, or achieving that a dataset can be used for publication even when initial statistical tests do not produce statistically significant results. However, it should be clear that interim analyses seriously break the rules of NHST and need correction for multiple testing (e.g., Wagenmakers, 2007; Simmons et al., 2011). Hence, especially when multiple researchers work together, they should encourage each other to correct for currently hidden multiple testing. In particular, co-authors should be able to challenge any indications of the techniques illustrated here: the responsibility for following a proper scientific process is shared.

Further, using *ad hoc* grouping variables and then publishing statistically significant results is clearly inadequate. Having many potential grouping variables is especially a danger in neuroimaging where a large number of brain activity measures can be correlated with and grouped by a large number of behavioral or other brain activity measures (see Kriegeskorte et al., 2009; Vul et al., 2009). Another special area of danger is the domain of ‘big data’ and very large databases in general which may enable several spurious analyses (Meehl, 1967; Lykken, 1968; Khoury and Ioannidis, 2014). Overall, in any studies with potentially very large number of variables and large volume of data (i.e., large power to detect small but irrelevant effects and the ability to select variables with occasionally appearing large but random effects) it is extremely important to clearly justify study objectives and optimally, to pre-register these objectives before the study is run (Simmons et al., 2011).

Crucially, the contents of the whole database (all variables and case numbers) a study is based on should be clarified even if most parts of that database may not be related to the published findings. Similarly, online supplementary material should present significant and non-significant correlations and/or relevant group differences along unpublished variables. These measures can help to avoid cherry picking statistically significant variables and/or group differences from large databases (see Simmons et al., 2011; Ioannidis et al., 2014 for more detailed recommendations).

## Author Contributions

DS has designed, carried out and written up the research.

## Conflict of Interest Statement

The author declares that the research was conducted in the absence of any commercial or financial relationships that could be construed as a potential conflict of interest.

## Footnotes

1 www.mathworks.com
